# Efficacy of Topical Silver Nitrate for Control of Post-tonsillectomy Hemorrhage

**DOI:** 10.7759/cureus.22857

**Published:** 2022-03-04

**Authors:** Eric Rohe, Malia Gresham, Rebecca Rohde, Lauren Cass, Jennifer V Brinkmeier, Adrienne Childers

**Affiliations:** 1 Otolaryngology - Head and Neck Surgery, University of Nebraska Medical Center, Omaha, USA; 2 Otolaryngology - Head and Neck Surgery, Saint Louis University School of Medicine, St. Louis, USA; 3 Otolaryngology - Head and Neck Surgery, Medical College of Wisconsin, Milwaukee, USA

**Keywords:** tonsil bleed, chemical cautery, silver nitrate, tonsillectomy, post-tonsillectomy hemorrhage

## Abstract

Objective

Management of patients with post-tonsillectomy hemorrhage (PTH) is not well defined but may include observation, topical bedside treatments, or return to the operating room. Data on the use and efficacy of silver nitrate as a topical bedside agent for the management of PTH remain unexplored. Our primary objective was to assess the efficacy of silver nitrate in reducing the need for operative control of PTH.

Methods

Single-institution retrospective chart review included patients aged 5-18 years who presented with tonsillar bleeding within 30 days of tonsillectomy. Patients undergoing observation or bedside silver nitrate cautery were compared based on clinical characteristics and experience of the physician performing the procedure. The outcome of interest was rebleeding requiring operative control. Sample characteristics according to treatment modality were described using Fisher’s exact tests and ANOVA.

Results

Of the patients eligible for inclusion, 29 (20%) were observed and 70 (48.3%) were treated with topical silver nitrate. Age was the only statistically significant clinical difference among treatment groups. The silver nitrate group had more patients who underwent operative control of PTH compared to the observation group (p = 0.004). When comparing the need for operative control between the observation group and patients who had initial success with silver nitrate, there was no difference (p = 0.29). No differences were found in the rate of bleeding requiring operative control when comparing experience of the physician performing the procedure (p = 0.20).

Conclusion

More patients who underwent silver nitrate cautery required PTH control in the operating room compared to the observation group. This may be due to patient selection as our results also suggest that there is no statistical difference in rates of operative control of PTH when comparing initial successful treatment with topical silver nitrate to observation. Age is likely a factor that was used by physicians in this study to decide the initial management of PTH. Provider experience does not appear to affect rebleeding rates. Future studies are necessary to evaluate the clinical impact of silver nitrate in the context of PTH and will benefit from more robust sample sizes and enhanced diversity in the sample group.

## Introduction

More than 500,000 tonsillectomies are performed annually in the United States in children younger than 15 years, making it the second most common pediatric surgery performed in the country [1,2]. Though tonsillectomy is generally considered safe, post-tonsillectomy hemorrhage (PTH) is a well-known complication. There are two types of PTH. Primary PTH occurs within the first 24 hours post-tonsillectomy, while secondary PTH can occur any time following the initial 24-hour post-operative period. Peak presentation of secondary hemorrhage is from post-operative days six to seven with a usual range from days two to 15, though presentation with secondary PTH up to postoperative day 21 has been reported [3,4]. Secondary PTH is more common than primary hemorrhage and may be considered more serious as the patient is often at home in a non-hospital setting [5].

The rate of PTH is often quoted to be 2-3%, but some have reported rates as high as 10% [1,3,4]. Of those who present with a complaint of PTH, about 9.4-13% may require major intervention such as return to the operating room and/or transfusion [3,4]. The majority of PTH presentations are self-limited or require only minor intervention [3]. Standardization of management is difficult due to the variety of definitions of PTH seen in the literature. These definitions can range from a convincing history of PTH with a normal oropharyngeal exam to significant active bleeding. A positive physical exam can also have a variety of meanings ranging anywhere from a clot in the oropharynx to significant active bleeding [4]. In addition to the variety of definitions of PTH, there is some debate as to whether operator experience can lead to variable rates of PTH. Some studies suggest that trainees have higher rates of secondary hemorrhage compared to consultants while others suggest there is no difference in secondary PTH rates when comparing physician experience [5,6].

The majority of patients presenting with active arterial or venous oropharyngeal bleeding will be taken to the operating room for control of hemorrhage. Management is less defined when it comes to patients with a normal oropharyngeal exam or a clot in the oropharynx without active bleeding [4]. In these cases, options can include observation, topical bedside treatments, or return to the operating room. Management is largely institution-dependent due to the lack of consensus in the literature [4].

The effectiveness of topical bedside treatments has largely been unexplored to this point. One study surveying practicing otolaryngologists found that 13% would attempt topical bedside treatment if a physical exam showed a clot in the oropharynx but no active bleeding and that up to 6% may attempt this treatment for an active tonsillar bleed [7]. Those rates increase to 45% and 38%, respectively, if the patient is a cooperative teenager [7]. One commonly used bedside treatment is silver nitrate. To our knowledge, there has never been a study assessing the effectiveness of topical silver nitrate use in the management of PTH. The primary goal of this study is to determine whether topical silver nitrate cautery treatment of PTH can reduce the need for returning to the operating room compared to observation. A secondary aim is to assess whether the experience level of the provider applying silver nitrate impacts the success of topical silver nitrate treatment.

## Materials and methods

**Figure 1:**
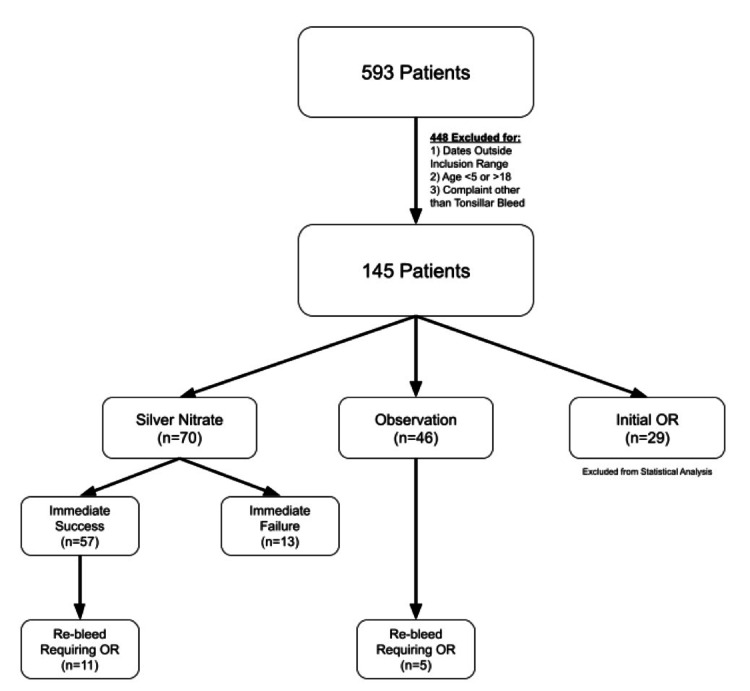
Flow sheet detailing how inclusion criteria were used to identify patients to be included in statistical analysis.

This was a Health Insurance Portability and Accountability Act (HIPAA)-compliant, retrospective, single-center cohort study. The study was reviewed and approved by the Saint Louis University Institutional Review Board (protocol 21906). Patients aged 5-18 years who underwent tonsillectomy with or without adenoidectomy (Current Procedural Terminology (CPT) codes 42820, 42821, 42825, and 42826) between December 2, 2013 and January 2, 2018 were identified. Among this group, patients who presented to the emergency department between January 1, 2014 and January 31, 2018 for tonsillar bleeding within 30 days of surgery were eligible for inclusion. The initial search by CPT codes yielded 6,037 patients. Results were filtered by patients returning to the emergency department from January 1, 2014 to January 31, 2018 and a list of 593 patients was produced. Of these, another 448 patients were excluded due to dates outside inclusion criteria (22 patients), patients' age outside inclusion criteria (46 patients), and presenting to the emergency department with a complaint other than tonsillar bleed (380 patients) (Figure 1). Chart review was performed on the included 145 patients to determine treatment method(s) utilized, as well as patient outcomes. Collected clinical characteristics of the patients include sex (male or female), indication for initial surgery (infectious concerns or sleep concerns), comorbidities (none, one, or more than one), family history of bleeding disorder (yes, no, or unknown), laterality of bleeding (left, right, bilateral, or unknown), location of bleeding within the tonsillar fossa (superior pole, mid-portion, inferior pole, multiple sites, or unknown), physical exam (active bleeding, clot present, or unknown/neither), and post-operative day at presentation (0-30). Treatment for PTH included admission for observation, bedside treatment with silver nitrate cautery, and control in the operating room. Observation and silver nitrate treatment groups were compared on the outcome of rebleeding requiring control in the operating room. The clinical experience of the person performing the procedure was categorized as junior resident (post-graduate year (PGY) 1-2) and senior resident (PGY 3-5). Sample characteristics according to treatment modality were compared using Fisher’s exact tests for categorical variables and ANOVA testing for continuous variables. Small sample sizes precluded further analyses of these data. A p-value of <0.05 was considered for statistical significance. Our local institutional review board approved the protocol and waived the informed consent requirement.

## Results

A total of 145 patients met the inclusion criteria. A total of 46 (31.7%) patients were treated with initial observation, 29 (20.0%) patients underwent immediate control of hemorrhage in the operating room, and the remaining 70 (48.3%) patients had bedside treatment with silver nitrate cautery. The mean age of the silver nitrate group was approximately three years older than the observation group (Table 1). No other clinical characteristics were associated with the treatment group. Fisher’s exact tests were not performed on variables of laterality of bleed, location of bleed, or physical exam due to large percentages of missing/unknown data in these groups.

**Table 1:** Patient characteristics. * Statistically significant.

	Silver nitrate (n = 70)	Observation (n = 46)	OR (n = 29)	P-value
Average age (SD)	11.3 (3.9)	8.8 (3.5)	8.8 (3.5)	0.0005*
Sex				0.14
Male	37.1	47.8	58.6	
Female	62.9	52.2	41.4	
Indication (%)				0.34
Infectious	44.3	30.4	41.4	
Sleep	55.7	69.6	58.6	
Comorbidities (%)				0.62
0	50.5	37	51.7	
1	32.9	39.1	34.5	
Multiple	17.1	23.9	13.8	
Family history of bleeding disorder (%)				0.065
No	87.14	89.1	79.3	
Yes	8.6	0	12.8	
Unknown	4.3	10.9	6.9	
Laterality (%)				--
Left	47.1	17.4	67	
Right	44.3	10.9	20.7	
Bilateral	8.6	4.4	10.3	
Unknown	0	67.4	0	
Location (%)				--
Superior	42.7	17.4	44.8	
Mid	24.3	4.6	27.6	
Inferior	32.9	2.2	13.8	
Multiple	0	0	3.5	
Unknown	0	76.1	10.3	
Physical exam				--
Active bleeding	24.3	6.5	24.1	
Clot	74.3	21.7	75.9	
Unknown/neither	1.4	71.7	0	
Postoperative day average (SD)	6.7 (2.7)	6.5 (4.0)	7.0 (2.5)	0.81

Among the 70 patients treated with silver nitrate, 13 (18.6%) failed initial bedside treatment and were taken immediately for operative treatment. Of the 57 patients with the initial control of bleeding after bedside silver nitrate, 11 (19.3%) patients experienced a rebleeding event that required operative control. In total, 24 (34.3%) patients treated with silver nitrate had immediate or eventual return to the operating room for control of hemorrhage. Five (10.9%) of the patients in the observation group experienced a bleeding event requiring operative control. There was a greater need for a return to the operating room among the silver nitrate group compared to the observation group (34.3% vs. 10.9%, p = 0.004). When comparing the need for operative control between patients who underwent observation and patients who had initial success with silver nitrate, there was no difference (10.9% vs. 19.3%, p = 0.29).

A total of 37 (52.8%) patients were treated with silver nitrate by junior residents and 33 (47.1%) patients were treated by senior residents. Rebleeding occurred in 21.6% of patients treated by a junior resident and 9.1% of patients treated by a senior resident (p = 0.20).

## Discussion

PTH remains a commonly seen adverse event with reported rates of occurrence ranging from 3% to 10% [1,3,4]. The majority of presentations remain self-limited or only require minor intervention, but a number of cases do require operative control of bleeding. To date, it has been difficult to standardize management due to various definitions of PTH in the literature as well as varying definitions of positive physical exam findings. Many patients presenting with active bleeding will be taken to the operating room, but that still leaves the question of how to manage patients who have a convincing history of hemorrhage with either a normal oropharyngeal exam or oropharyngeal clot.

Silver nitrate cautery is a common intervention used to control epistaxis originating in the anterior nasal cavity. The chemical reacts by releasing free radicals, which leads to the oxidization of organic tissues [8]. This forms an eschar that obstructs and scleroses blood vessels [9]. Prior studies have shown that failure rates of silver nitrate can be quite high, ranging from 16% to 26% [10,11]. No serious adverse effects have been associated with nasal silver nitrate cautery, but pain is often reported [8]. Silver nitrate cautery is also a reported topical therapy to control PTH [12,13]. A cooperative patient is essential, as this treatment requires the examiner to apply a painful silver nitrate treatment to the sensitive and reactive oropharynx. Though this intervention is frequently reported, we have not found any prior studies aimed at identifying the effectiveness of silver nitrate in controlling PTH.

As mentioned earlier, proper patient selection for bedside silver nitrate treatment is paramount. The ideal patient would be calm, cooperative, and would require a positive physical exam finding of either clot or active bleeding to help direct the precise site of cautery application. The studied age range was selected in an attempt to maximize the possibility of encountering patients with these characteristics while also obtaining an adequate sample size. The average age of the silver nitrate group was approximately three years older than the observation and immediate operative treatment groups, suggesting that providers may be more likely to attempt a bedside procedure in older children. This would be consistent with previous surveys of practice trends among practicing otolaryngologists [7]. It remains unclear whether there are other clinical features of an older child’s presentation that may have made providers in this study choose silver nitrate for initial management.

Our study sought to determine the utility of topical silver nitrate in reducing the need for operative control of hemorrhage when compared to observation. A higher percentage of patients in the silver nitrate group underwent control of hemorrhage in the operating room compared to the observation group. However, excluding the portion of the silver nitrate group that immediately failed treatment, there is no significant difference in rates of control in the operating room between this subgroup and observation. This suggests that silver nitrate may be a way to avoid some returns to the operating room if it does not immediately fail.

When further reviewing patient characteristics, one finds that 98.6% of patients in the silver nitrate cohort had a physical exam finding of active bleeding or oropharyngeal clot (24.3% and 74.3%, respectively). Conversely, only 28.2% of patients in the observation group were noted to have findings of active bleeding or oropharyngeal clot (6.5% and 21.7%, respectively). Subjects in the observation group were noted to have a physical exam finding in the unknown/neither category, which includes a normal physical exam, in 71.7% of cases. Though seemingly minor, this could represent two slightly different patient populations in whom separate treatment considerations should be made.

Major limitations to this study include its retrospective nature and relatively small sample size. Further, studies with more robust sample sizes are necessary to help determine the optimal management strategy for patients with PTH and whether or not there is an ideal patient population who may benefit from treatment with topical silver nitrate.

## Conclusions

The use of silver nitrate in the pediatric PTH population remains an understudied topic. A higher percentage of patients in the overall silver nitrate group underwent control of hemorrhage in the operating room compared to observation. Our results also suggest that there is no statistical difference in rates of rebleeding that require operative intervention when comparing initial successful treatment with topical silver nitrate to observation. These data could be affected by our relatively small sample size and the retrospective nature of the project. Age is likely a factor that was used by physicians in this study to decide the initial management of PTH. Our results also suggest the experience level of the provider applying topical silver nitrate does not affect rebleeding rates. Future studies are necessary and important to evaluate the clinical impact of topical silver nitrate in the context of PTH and will benefit from more robust sample sizes and enhanced diversity in the sample group.
